# The challenges and benefits of a genuine partnership between Music Therapy and Neuroscience: a dialog between scientist and therapist

**DOI:** 10.3389/fnhum.2015.00223

**Published:** 2015-05-01

**Authors:** Wendy L. Magee, Lauren Stewart

**Affiliations:** ^1^Music Therapy Program, Boyer College of Music and Dance, Temple UniversityPhiladelphia, PA, USA; ^2^Department of Psychology, Goldsmiths, University of LondonLondon, UK

**Keywords:** neuroscience, music therapy, debate, interdisciplinary, patients, clinical protocols

## Abstract

Collaborations between neuroscience and music therapy promise many mutual benefits given the different knowledge bases, experiences and specialist skills possessed by each discipline. Primarily, music therapists deliver music-based interventions on a daily basis with numerous populations; neuroscientists measure clinical changes in ways that provide an evidence base for progressing clinical care. Although recent developments suggest that partnerships between the two can produce positive outcomes for both fields, these collaborations are not considered mainstream. The following dialog between an experienced professional from each discipline explores the potential for collaboration, as well as the misconceptions that may be preventing further synergies from developing.

Two professionals from different sides of the neuroscience and music therapy debate present an informal dialog exploring realities and beliefs that have benefited or hindered collaboration. As a music therapist who has turned to neuroscience for evidence in neurological rehabilitation clinical practice, and a neuroscientist who has been motivated by the implications of her research for clinical populations, we present this dialog in an interview format. This format was chosen to encourage genuine questioning and exploration of issues that are implicit to potential collaborations, and which remain unexplored in empirical research.

**WM:** Lauren, in your view, how can music therapy contribute to the wider perspective of clinical practice and research?

**LS:** I think there is no question that the properties of music, in terms of intrinsic features, as well as the potential for engagement, emotional response and interpersonal communication, can be very powerful across a range of clinical situations. When used appropriately, music is ethically acceptable, side-effect free, can be intricately tailored to personal preferences and tastes, and in some cases may provide a cost-effective alternative to pharmacological sedation (Loewy et al., 2006). Exploiting the potential benefits of music is not only essential for advancing clinical practice, but also in elucidating and characterizing how music acts on the brain. There is much to be gained from a joint enterprise where practice and research can reciprocally inform one another.

But achieving such collaborations takes time: How do you think our respective disciplines are doing in this regard, Wendy? Are you sensing a significant productive collaboration in recent years **WM:** I think there are many interesting collaborations emerging that illustrate how a genuine partnership between the two professions can draw on the strengths of each to benefit research and improve clinical practice. One example is the new MANDARI collaboration (music and the neurodevelopmentally at risk infant) which has brought together researchers and clinicians from diverse disciplines to discuss the potential of music at the earliest possible state in life (http://www.gold.ac.uk/mandari/). The different disciplinary languages and frameworks are explicitly discussed to permit a platform for genuine interdisciplinary engagement, including scholarly critique of frameworks and assumptions that may be implicitly entrenched in our respective disciplines.

A number of studies also provide models for collaborations between the two disciplines. To take just a few examples: Thaut et al. (2005) examined music as a mnemonic device for learning and memory with Multiple Sclerosis patients and its effect on neuronal synchrony; Särkämö et al. (2008) examined the impact on cognitive recovery, mod and brain activation following stroke and O'Kelly et al. (2013) explored brain responses to music in patients with disorders of consciousness who cannot show behavioral responses. Studies such as these demonstrate the potential of a combined music therapy/neuroscience approach to give insights into “how” music works and “why” we see clinical improvements. The knowledge that stems from such collaborations ultimately has the potential improve interventions offered to patient populations.

However, I personally feel that the potential synergies between our two fields have yet to realize their full potential. I've been working in music and neurology for around 25 years and certainly I've wanted to engage with neuroscientists to a greater degree, particularly through my work with complex, brain-damaged populations. As a clinician, I have found reading the neuroscience literature invaluable for drawing out relevant information in order to both inform my own understanding of the brain and, where possible, apply it in an evidence-based way in practice with clients.

Personally, I have been able to build relationships with individual neuroscientists where we have a common interest in clinical populations. However, these relationships have not been able to develop in more systematic ways. We largely read different journals, go to different conferences and belong to different societies. Although music therapists are increasingly attending more neuroscience-based conferences and publishing in neuroscience journals, there is very little infrastructure to allow these two disciplines to interact in ways that can reciprocally inform each other. Perhaps you have thoughts on how we might advance collaborations and dialog? What do you feel has been a barrier to collaborations to date?

**LS:** As you say, there are enormous challenges to interdisciplinary working, which is easy to express support for but more difficult to realize! My recent involvement with the MANDARI collaboration showed me that not only do we speak very different languages but we also have very different motivations for our involvement, and what counts as an interesting question or goal for one person, can seem less important to others. It's hard to articulate our deep-seated motivations, but an honest exchange of where each party is coming from is vital to ensure people are not pulling in different directions without even realizing it.

Added to this is the fact that many areas of clinical practice might remain hidden to the research community, since many clinicians do not have the time or resources to conduct or publish research. They might communicate it within their local practice-based networks only. This can provide a skewed picture of what is actually going on clinically, which often does not reflect the breadth of practice and associated theories and frameworks that are being used.

Special initiatives, such as this Frontiers issue, can provide a platform for knowledge exchange, as can seeking out opportunities to understand more about the very different worlds each of us inhabit. But ultimately, the most productive collaborations will be motivated by individuals who have a vision of how research and practice can complement one another, and who work from a grass roots level to make it happen.

Perhaps we could consider the different kinds of motivations that typically drive clinicians vs. researchers—what are your thoughts on that?

**WM:** A primary motivation of a music therapist is to improve clinical methods in order to benefit the patient. Therapists are very much at the coalface, working with people who do not have straightforward types of pathologies; this is typical in catastrophic brain injury. They do not have neat lesions in one area of the brain, they have complex problems, and they're all different.

For music therapists, the drive to do research is prompted by what happens in the therapy room during the clinical intervention. Therapists are interested in questions about “what is it that works?” and “which process works best for that patient?” Often they work so closely with the patients and their families, they have difficulty in standing back and looking at the bigger picture, which is necessary for a researcher. Lauren, do you feel this is a barrier for neuroscientists engaging with the music therapy profession in research collaborations? Perhaps it is easier for neuroscientists to do this, since they are less engaged in directly working with patients?

**LS:** As you say, one of the important issues for music therapists, is obviously the individualized, tailored approach, while, for researchers, group designs where an intervention can be implemented in the same way across a group of patients, is often preferred. This may involve abstracting something personal and bespoke into a “one size fits all” approach that may, in the end, turn out to be less relevant and less effective for the patient group. So there's a tension between an intervention, which may be idiosyncratic and highly personalized from one patient to the next, with the need for a design that incorporates standardization and replicability. It's possible to have a design that incorporates a tailored approach, and can be analyzed in a statistically robust way, but such an approach is not orthodox for most neuroscientists.

**WM:** Indeed. I should add, the type of well-controlled protocols that neuroscientists are used to challenge real-world settings on two fronts. First, if a protocol does not meet a patient-centered need that the patient or the therapist feels is most important (e.g., an emotional need over a functional need such as hand grasp), then the clinician and the patient lose motivation to continue. There are also ethical questions about using protocols that are not best suited to patient needs. Second, music is a medium that provides opportunities for spontaneity and play, which are both important features in therapy, learning and rehabilitation. These features can be challenging to incorporate into a controlled protocol.

Music Therapists in recent years have become more involved in research to generate evidence, particularly with randomized controlled trials (RCTs), which are considered one of the highest forms of “evidence” in health care. RCTs are challenging on a number of fronts; one of which concerns the difficulty of formalizing the intervention in terms of a standardized protocol. We know that this is one of the criticisms that neuroscientists have of Music Therapy. Ultimately, therapists have been trained to view each client as an individual, and tailor intervention to that individual. Adopting standardized protocols can be seen as not taking account of individual differences and treating that person as a unique being.

This is one reason why RCTs are difficult to do in practice and are rarely the best method for getting at complexity, for instance, researching rehabilitation after catastrophic brain injury where single-subject designs are more suitable. But, on the other hand, if we completely reject the notion of RCTs altogether, we risk missing the opportunity to engage in testing out the efficacy of music therapy interventions, using research designs that are widely recognized as the “gold standard” in health care. An alternative is to do an RCT where protocols are defined in a way that enables flexibility. For example, one protocol, which has been written for working with children with Autism spectrum disorders, defines a complex intervention of improvisational Music Therapy (Geretsegger et al., 2012). This is a challenging intervention to protocolize as it draws on musical spontaneity and play to improve specific non-verbal communicative behaviors typical with this population. The protocol manages to describe the intervention procedures with enough precision to enable a trained therapist to deliver the intervention but also allows for spontaneity in response to the client's musical and communicative behaviors.

**LS:** Another example of an RCT, that has a flexible implementation, can be seen in study where parents were trained to deliver live Music Therapy in the neonatal intensive care unit (Loewy et al., 2013). Although the parents had been trained along broadly similar lines, the detail of delivery was rather different. So you don't always need to disregard the lived experience when you are doing research, you just need to be a bit clever about it.

In relation to this, I'm aware that for most scientists, the Cochrane Reviews (http://www.thecochranelibrary.com/) would be the first port of call in trying to establish whether Music Therapy was deemed effective for a particular clinical group. With their reliance on RCT designs, is there a danger that some high quality Music Therapy studies are being overlooked?

**WM:** The Cochrane Reviews are considered the “gold standard” and they evaluate all the quantitative research that has taken place on an intervention with a specific population, e.g. Music therapy for Acquired Brain Injury (Bradt et al., 2010). However the inclusion criteria used to evaluate research studies are very narrow. This means that many studies that present a compelling argument for the effectiveness of Music Therapy in a certain clinical context are excluded from the “evidence base.” The Cochrane's evaluative criteria include principles of randomization, allocation concealment and double blinding in order to minimize or eliminate bias completely. These designs are modeled on principles of testing pharmaceuticals, which is not the best application for many therapeutic interventions. As an author of a Cochrane review, I think that it is really important for us to engage with the evidence debate.

**LS:** In our discussion so far, we have yet to touch on the distinction between Music Therapy and Music Medicine. Could you outline how those two approaches differ?

**WM:** Music Medicine involves interventions using music that have a clinical outcome in mind, but where the outcome is not reliant on the relationship between the client and the person giving the intervention. That is, the intervention does not rely on some type of human musical dialog and relationship development (or process) that is typical in a therapeutic interaction. These interventions are typically implemented by nurses, doctors and even dentists. The interventionist could simply leave the music with the client. A good example of this is the management of pre-operative pain and anxiety, where a patient is given recorded music to listen to. I believe there is a role for non-complex music interventions such as these, where there is minimal risk to the patient and can be delivered by a wide range of health professionals. Such interventions do not require training in how to deliver the intervention, or in how to enhance the interpersonal interaction or analyze the patient's responses. This contrasts with clinical scenarios that do require complex interventions. Some examples of these might be psychological difficulties where the person has trouble in developing or maintaining interpersonal relationships, due to Autism spectrum disorders, an attachment disorder, or is dealing with the psychological trauma caused by bereavement, loss or abuse. These clinical needs demand a human element: another person to work with the client in order to provide them with the experience of relearning to “relate.” These clinical needs demand very different musical and therapeutic interventions to simply playing a patient recorded music.

**LS:** So in some cases, is music used as a framework to facilitate a more standard type of talking therapy?

**WM:** Relationship development, through the use of music, is certainly comparable to speaking therapies. Music can be a useful medium to work on interpersonal issues for a number of reasons. Within a musical interaction, you can sing “with” a person, not simply sing “at” or “to” one another; you improvise, listen, attune and respond using imitation or reflection. With some populations it is more effective than communicating with words, particularly for those who may find it difficult to speak or perhaps those who have not yet acquired language or have lost language due to brain damage.

**LS:** I sometimes think that the skills and knowledge that music therapists have are not well understood, from the perspective of the basic science researcher. For instance, at a recent talk I attended, the presenter who was a non-clinician scientist, was asked whether the described intervention given to a particular clinical group was administered by a music therapist or not. The response was “No, but the person delivering it was a competent musician.”

**WM:** Yes, this is important to articulate. In some clinical settings, the assumption may be that a music therapist is there to simply entertain the patient in order to lift their mood. In fact, music therapists are professionals who have been trained to a high standard musically, but more importantly, they have been trained to work with clinical populations and to use music in ways to address a wide range of social, emotional, behavioral and physical needs. Most importantly, they are trained in attuning to other people, musically and emotionally, whilst maintaining strong boundaries between themselves and the client.

Simply learning a protocol through reading a theoretical research paper and attempting to apply it within a clinical setting presents many risks to the patient and the person doing the music protocol. When working with clinical populations, unexpected difficulties can arise whereby an untrained person may not be able to manage the situation, (e.g., extreme agitation, distress, physical self-harm), and interact with the patient safely. A music therapist has skill and expertise to a recognized standard in assessing a situation and adapting a protocol to a clinical situation.

**LS:** Perhaps one of the difficulties in understanding what music therapists do comes from the existence of several different approaches and philosophies within the profession. The kind of Music Therapy that is probably most familiar to neuroscientists is Neurologic Music Therapy (Thaut, 2005), but in music therapy circles, many other “flavors” are dominant and some of them seem to downplay functional goal-setting, which to neuroscientists, can be difficult to appreciate—could you comment on that?

**WM:** I think this point you bring up is a really important issue. As with other professions (e.g., Psychology) there are different theoretical models in music therapy that range from behavioral, to psychodynamic, to music-centered, to humanistic and so on. Each approach has its own merits and some will be more suited to certain contexts than others. However, the important thing is that the model of music therapy used is appropriate to the patient's needs, and the therapist can articulate the outcomes and rationale behind the method they are using in ways that the patient, families and colleagues can understand.

**LS:** We've covered a lot of ground here, but I wonder if I can finish up by asking you where you see Music Therapy making the biggest inroads going forward?

**WM:** I feel very excited about interdisciplinary collaborations such as that modeled by MANDARI, because these have big implications for both of our professions, and most importantly, for patient care. Interdisciplinary research with other clinical professions (e.g., nursing; medicine) is also growing and will improve research through accessing more participants who are suited to studies. Research that continues to explore music's impact on the brain with clinical populations is also a priority so that we can develop interventions that will have greatest impact, particularly when we consider Dementia and Stroke as the two largest and fastest growing populations in societies around the globe. We need to understand why and how music works and refine interventions. Tapping into populations for which we have no evidence base is also a priority, such as post-traumatic stress disorder, particularly those who have returned from military conflict and the devastated populations left after conflict or torture. Music Therapy's impact in this domain would be relevant both for neurological rehabilitation but also the psychological trauma that cannot be explored easily using verbal interactions. The findings potentially would be relevant for a number of populations where psychological trauma is a major factor.

## Conflict of interest statement

The authors declare that the research was conducted in the absence of any commercial or financial relationships that could be construed as a potential conflict of interest.

## References

[B1] Bradt J. Magee W. L. Dileo C. Wheeler B. McGilloway E. (2010). Music therapy for acquired brain injury. Cochrane Database Syst. Rev. 7:CD006787. 10.1002/14651858.CD006787.pub2 20614449

[B2] Geretsegger M. Holck U. Gold C. (2012). Randomised controlled Trial of Improvisational Music therapy's Effectiveness for children with Autism spectrum disorders (TIME-A): study protocol. BMC Pediat. 12:2. 10.1186/1471-2431-12-2 22221670 PMC3280156

[B3] Loewy J. Hallan C. Friedman E. Martinez C. (2006). Sleep/sedation in children undergoing EEG testing: a comparison of Chloral Hydrate and Music Therapy. Am. J. Eletron. Technol. 46, 343–355. 10.1016/j.jopan.2005.08.001 17285817

[B4] Loewy J. Stewart K. Dassler A. M. Telsey A. Homel P. (2013). The effects of music therapy on vital signs, feeding, and sleep in premature infants. Pediatrics 131, 902–918. 10.1542/peds.2012-1367 23589814

[B10] O'Kelly J. James L. Palaniappan R. Taborin J. Fachner J. Magee W. L. (2013). Neurophysiological and behavioral responses to music therapy in vegetative and minimally conscious states. Front. Hum. Neurosci. 7:884. 10.3389/fnhum.2013.00884 24399950 PMC3872324

[B9] Särkämö T. Tervaniemi M. Laitinen S. Forsblom A. Soinila S. Mikkonen M.. (2008). Music listening enhances cognitive recovery and mood after middle cerebral artery stroke. Brain 131, 866–876. 10.1093/brain/awn013 18287122

[B6] Thaut M. (2005). Rhythm, Music, and the Brain: Scientific Foundations and Clinical Application. New York, NY: Routledge.

[B8] Thaut M. H. Peterson D. A. McIntosh G. C. (2005). Temporal entrainment of cognitive functions: musical mnemonics induce brain plasticity and oscillatory synchrony in neural networks underlying memory. Ann. N. Y. Acad. Sci. 1060, 243–254. 10.1196/annals.1360.017 16597771

